# Assessing the impact of draught load pulling on welfare in equids

**DOI:** 10.3389/fvets.2023.1214015

**Published:** 2023-08-17

**Authors:** Syed S. U. H. Bukhari, Rebecca S. V. Parkes

**Affiliations:** ^1^Department of Veterinary Clinical Sciences, Jockey Club College of Veterinary Medicine and Life Sciences, City University of Hong Kong, Kowloon, Hong Kong SAR, China; ^2^Centre for Animal Health and Welfare, Jockey Club College of Veterinary Medicine and Life Sciences, City University of Hong Kong, Kowloon, Hong Kong SAR, China

**Keywords:** donkey welfare, equine behavior, equine welfare, horse welfare, limb biomechanics, cart pulling, mule welfare, equine physiology

## Abstract

About 112 million working equids are the source of income for 600 million people globally. Many equids are used for pulling loads (up to 15,000 kg per day) to transport goods. Most of them are associated with brick kilns, mining, and agriculture industries in developing countries. They may suffer from welfare issues such as overloading, being beaten, and being forced to work for long periods. These issues may occur due to a poor understanding of load-pulling equids. Understanding their capabilities and the elements that influence them is critical for efficient performance and welfare. The measurement of stride characteristics and gait kinematics can reveal loading adaptations and help identify loading limitations. It is known that both loading and fatigue change the locomotor patterns of load-pulling horses. Heart rate is a stress quantifying metric and an important representative of the speed of work and draught force. Heart rate variability is a regularly used statistic to quantify a physiological response to stresses, but it has never been used for load-pulling equids. Changes in blood lactate, nitrogen, oxygen, and carbon dioxide contents are reliable biochemical indicators of the effects of load pulling. Changes in plasma cortisol levels reflect the intensity of exercise and stress levels in horses while pulling a load. However, eye blink rate is a cheap, simple, and immediate indicator of acute equine stress, and we suggest it may be used to aid in load-pulling equine welfare assessment. However, further research is needed for a standardized and evidence-based draught load pulling capacity of working horses, mules, and donkeys.

## 1. Introduction

**Figure 1:**
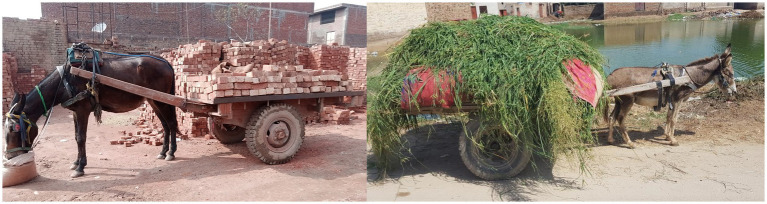
Draught load pulling mule in a brick kiln production system (left) and fodder cart pulling donkey in a rural area (right) in Pakistan. Photo: Syed S. U. H. Bukhari.

The global equine population is approximately 116 million (1), and out of this, 112 million are working equids (2). Working equids are the source of income for their owners (3) and help to sustain 600 million people globally (4), most of whom live in poor and marginalized communities (5). Working horses, mules, and donkeys are vital to people’s economic and social well-being (4). Carts hauled by horses, mules, and donkeys are essential modes of transportation in most of these communities, and carting is a source of income for a large proportion of the population in developing nations (Figure 1) (6). In low to middle-income countries (LMICs), while motorized transportation has grown quickly during the last few decades, the usage of equid’s power to pull carts for local transportation of goods has remained unchanged (7). Carts are used to transport building materials, commercial produce, and garbage (2). Equids are crucial in the growth of agriculture and other activities as they provide power for plowing and traction, playing an important role in the local economy (8, 9). Equids’ social and economic contribution to rural earning can be direct (providing transportation services) or indirect (plowing the soil to obtain farm products) (10). Therefore, underestimating their contribution could have a negative impact on society (11), as they perform domestic tasks as well as agronomic and local transportation (12). Working equids are sometimes a person’s only income source. They rely on them for day-to-day activities, providing access to medical care, access to schooling, and basic commodities to some of the globe’s most marginalized communities (13, 14).

**Figure 2:**
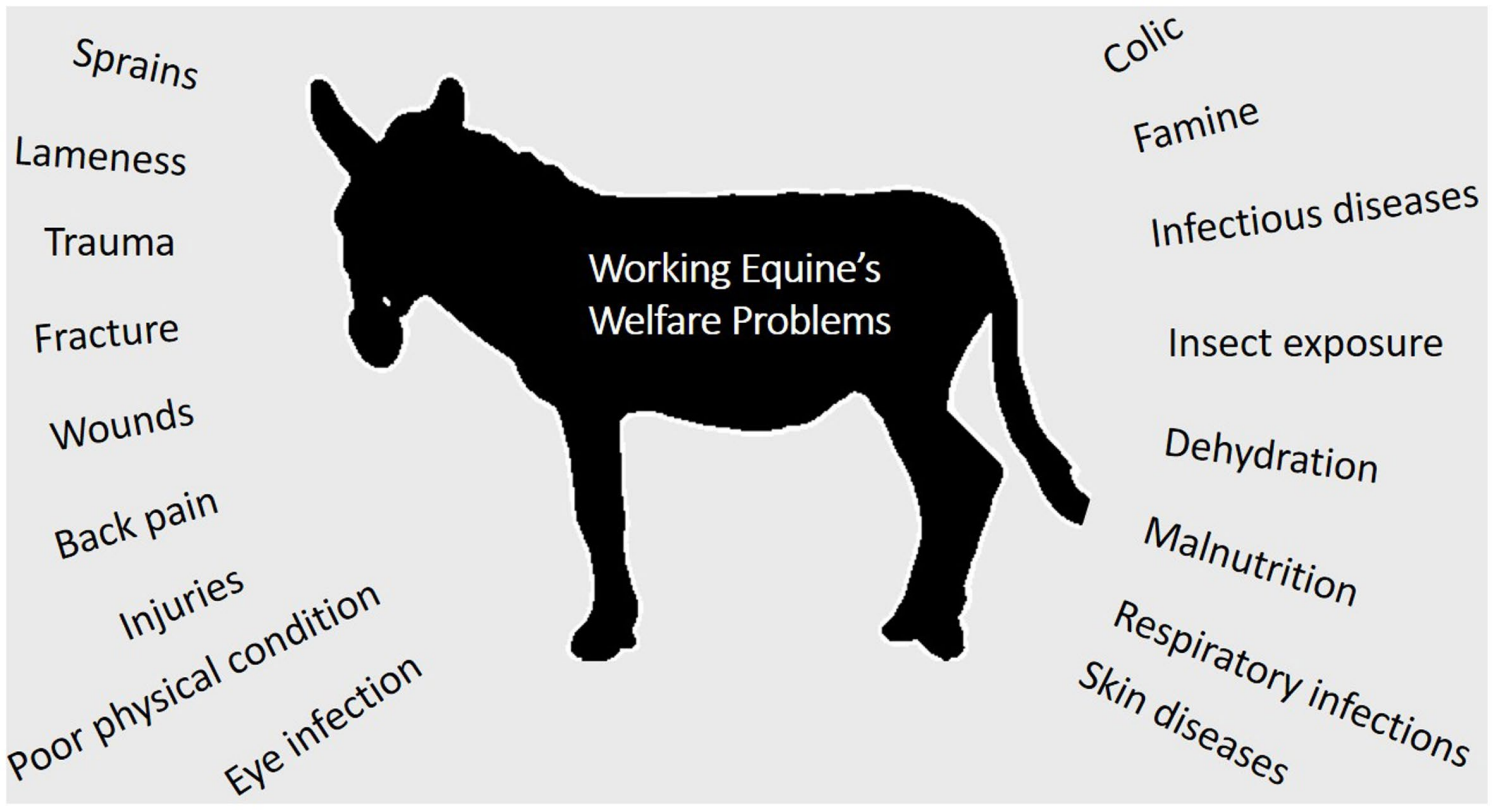
Common welfare problem of equids associated with draught load work.

The welfare standards of working equids remain inadequate in LMICs (13, 15). Eye infections, infectious diseases, colic, skin diseases, poor physical condition, respiratory infections, back pain, injuries, exhaustion, wounds, malnutrition, famine, fracture, heat stress, dehydration (2, 10), trauma, insect exposure (16), sprains, lameness, as well as other catastrophic injuries (15, 17) are some of the most common welfare issues (Figure 2). They are prone to locomotor system diseases (18), which become even more common when subjected to hazardous working situations (19). Lesions caused by inadequate harnessing, dehydration, foot and shoeing issues, poor body condition score, and behavioral issues such as aggressiveness are the most common welfare issues observed in working equids (11). Donkeys often have a lower welfare standard than horses, with the most common issues being poor physical condition and injuries (3). This could be due to the fact that horses are more valuable and sell for a higher price. Over half of working equids endure starvation, fatigue, illness, and injuries during their working lifetimes, often exacerbated by a lack of accessible and cheap animal health treatments (20). Most equids have limited access to veterinary care, and most illnesses go untreated. The demise of an equid or even a reduction in the time it is able to work causes many difficulties for the community it serves (21). It is essential to ensure that each animal is pain-free, injury-free, and disease-free by providing prompt diagnosis and medical care (20).

Welfare problems of working equids are not limited to physical ailments; many working equids have behavioral issues, including fear of humans and sometimes even despair (22). Beating donkeys is one of the major causes of behavioral problems. Beating a donkey not only causes wounds and physical pain but it also induces fear and severe stress to the animal (23). Their poor welfare is connected to difficult operational conditions and handlers who lack basic knowledge of general husbandry and effective working equid care, such as management of wounds, harness fitting and care, appropriate shelter arrangements, watering, veterinary services, and nutritional requirements (12). Donkeys and mules differ from horses in their behavior and require greater patience. If behavioral standards used to assess horses are used, their stoicism makes it more difficult to spot and diagnose problems in donkeys and mules (24). Working horses, mules, and donkeys, particularly in developing countries, must be considered in national livestock policy and programming (25).

The traction power of equids is used in brick-making industries in many LMICs (26). The work of horses, mules, and donkeys involves carting wet and dry bricks within brick kilns and from brick kilns to various places for use in the construction sector (10). In Egyptian brick kilns, donkeys are generally overloaded and may pull a cart averaging 2,040 kg in addition to the weight of the handlers, while suffering from pain and open lesions (12). In some LMICs, mules pull a draught load of about 1,500 kg per cart during a single trip, and there are about 8–10 trips per day (27), i.e., they pull about 15,000 kg per day. Moreover, equids making less frequent trips are 2.5 times more likely to carry heavier loads (28). Usually, a donkey (weighing 150–250 kg), working with brick kilns, transports 4,200 bricks (10,500 kg draught load) per day (29). Equines are frequently subjected to overwork and are regularly forced to work all day. Overworking is the cause of high prevalence of lameness in the young population of mules. This could be the reason of high turnover rate of working equids, with only 20% of animals owned for longer than 3 years (28). The most common concern is overloading, which exposes the animals to various wounds and back sores. The saddle and harnessing materials are frequently inappropriate, increasing the risk of equines suffering health and welfare issues (4). These issues may occur due to a poor understanding of the equid’s needs or the owner’s economic constraints.

Draught horses’ physical work demands strength for pulling and endurance for prolonged labor. Endurance may be defined as the ability to perform a muscular activity at a high level of intensity for extended periods (30). It is important to understand the impact of draught load pulling on working animals, but many equestrian sports also rely on draught load. Harness racing is a prominent horse racing sport that evolved from a historic, recreational sport during which horses compete at a set gait while pulling a two-wheeled cart (31). Trotting and pacing are the two different gaits used in harnessed races. A trotter moves its legs forward in diagonal pairs (right front and left hind, then left front and right hind striking the ground simultaneously), whereas a pacer moves its legs laterally (right front and right hind together, then left front and left hind) (32). Horses were driven long before they were ridden, so driving is the oldest competitive equestrian sport. It is still alive and well in the twenty-first century. In competitive carriage driving, drivers sit in a vehicle drawn by a single horse, a pair of horses, or a team of four horses and compete in three events: dressage, marathon, and obstacle driving (33). Horses may also pull a heavy load in competition, for example, heavy horse pull competition at the Calgary Stampede (33, 34). These sports have significant economic benefits for society (34).

Load pulling equids are of great value as they are used both as working equids in LMICs and in harness competitions internationally. However, most research focuses on ridden horses. People who use horse power should be aware of their limitations to maximize equine welfare. Understanding equines’ labor capacities including their load pulling abilities (how much they can/should pull?), which might influence their optimum field performance, is critical to their efficient utilization. Quantified load pulling limits could then be used by non-governmental organizations (NGOs), policymakers, and other stakeholders working with vulnerable communities and working equids to limit excessive load pulling and improve animal welfare. The biomechanical, physiological, biochemical, and behavioral impacts of pulling load on equids are discussed in this review.

## 2. Biomechanical assessment

**Figure 3:**
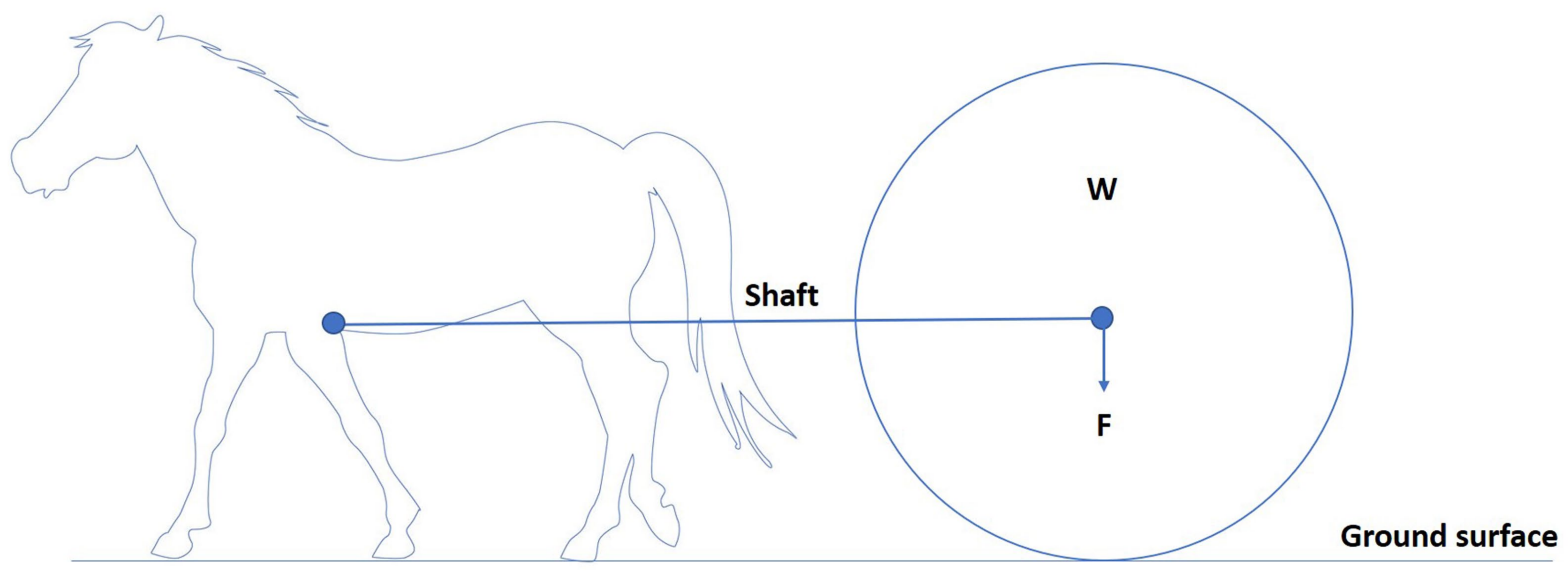
Horse pulling a load (W) with a zero-draught angle as the shafts are parallel to the ground. The arrow indicates the direction of the load’s force (F). The horse only needs to exert a horizontal force to move the load.

**Figure 4:**
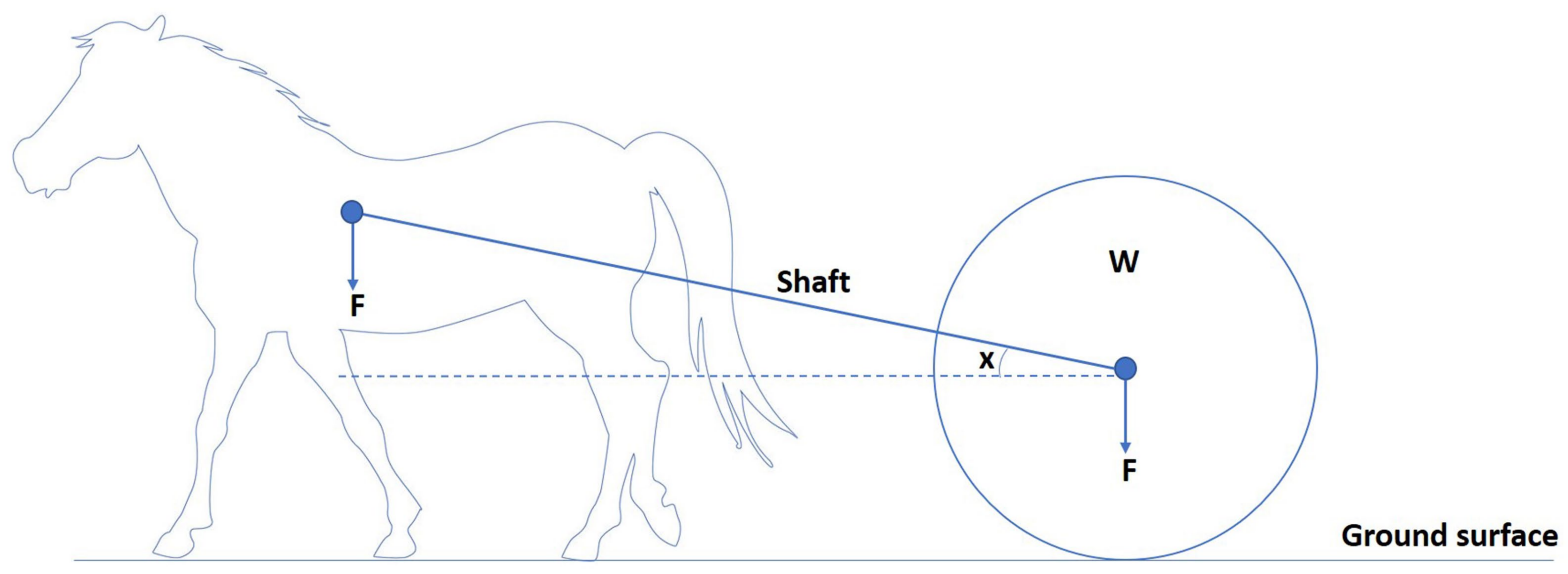
Horse pulling a load (W) with a draught angle (x) as shafts is not parallel to the ground. The arrow indicates the direction of the load’s force (F). The horse needs to exert both horizontal and vertical force to move the load, as the load is pulling back and down on the horse.

To understand the biomechanical effects of load pulling on equines, a basic understanding of the mechanics of load pulling is needed. Draught force can be defined as the force required to pull a load in the same direction of travel as the horse (30, 35). Horses pulling loads experience different forces. With a draught angle of zero and shafts parallel to the ground, the horse only needs to exert a horizontal force to move the load (Figure 3) (36). When there is a draught angle, the shafts are at an angle to the ground, and the horse must exert the same horizontal force and a vertical force because the load is pulling back and down on the horse (Figure 4) (36). This is related to the observation that horses can move faster, pulling rather than carrying a given load at a given gait (17). Therefore, horses dragging loads are exposed to different forces and are prone to a different set of injuries than horses carrying loads (36). The biomechanics of equine load pulling is not well studied. At the start of work, when horses are initially loaded by the horizontal-pulling load, their general movement pattern remains unchanged. More drastic variations in the movement pattern occur due to fatigue (37).

The measurement of stride characteristics and gait kinematics can reveal loading adaptations and help identify loading limitations (17). It is known that both loading and fatigue can change a horse’s locomotor pattern (37). In general, changes to stride patterns with speed are conserved across breeds; both Thoroughbred and draught horses tend to increase speed by increasing stride length more than stride frequency (38). When pulling a draught load, however, an increase in stride frequency and a decrease in stance time are seen (37). Stride frequency increases from 108.2, 105.4, and 108 strides/min to 117.2, 118.8, and 119.6 at the same speed of 9 ms^−1^ with 0.1kN, 0.2kN, and 0.3kN draught force, respectively (39). At similar canter speed (8 ms^−1^), stride frequency is greater in Thoroughbred than in draught horses, as mean stride frequency remains at 110.4 and 100.8 strides/min, respectively (38). Interestingly, during incremental (0.2 kN increased every 2 min) draught force exercises starting from 0.04 kN, stride length remained constant and did not change (40). In contrast to this, in another investigation, horses did reduce their stride length (from 3.74 to 3.65 m) in response to increased pulling load (0–34 kg) (37). Furthermore, stride length increases from 3.1, 3.5, and 3.1 m to 4.1, 4.6, and 4.1 m with a draught force of 0.1kN, 0.2kN to 0.3kN at the same speed of 9 ms^−1^ in load pulling horses, respectively (39). However, while slow trotting (3 ms^−1^), stride length is determined by the speed regardless of increasing weight resistance (40).

Comparing Thoroughbred and draught horses, the walk to trot transition is about two ms^−1^ in both breeds (38). The transition from trot to canter is between 4–6 ms^−1^ for Thoroughbred and 6–8 ms^−1^ in draught horses with draught force of 0, 5, 10, 15, and 20% of their body weight, respectively. However, changing the draught force does not affect gait type at any speed (38). As at a given pace, higher draught force is associated with increased stride frequency and shorter stride length. When we increase speed without a draught force and change force at a constant pace, the stride characteristics of Thoroughbred and draught horses are comparable (38).

The period of time when the foot is in contact with the ground’s surface is known as stance time (17). When pulling a load, horses reduce stance time in both forelimb and hindlimb (37). Horses reduce their stance time (from 0.165 to 0.157 s) in response to an increase in pulling load (from 0 to 34 kg), in contrast to horses under mounted, load, which increase their stance duration (17, 41). This disparity between load pulling and mounted load could be attributed to differences in vertical ground reaction forces or limb load, but this has not been well investigated in horses, mules, and donkeys. This could be because, theoretically, a draught angle of zero (shafts parallel to the ground) requires the horse to exert just a horizontal force to move the load (36); therefore, the load should not cause an increase in vertical ground reaction force on the limbs.

A walking horse (weighing 648 kg) with a speed of 2.11 ms^−1^, pulling 1,892 kg load for 4 h, generates a draught force of 0.59kN and produces a work of 15.69 MJ (42). Horses work more quickly on the first day of their work after some rest; during later days, they become slower. Compared to buffaloes or oxen hauling carts over a flat surface, a horse’s average pace is twice as fast (43, 44). Horses weighing 675–860 kg can constantly work at a rate of 0.75 kW for 10 h a day and travel a total of 32.2 km per day without becoming fatigued (42). While measuring time-averaged draught force (TADF) and distance-averaged draught force (DADF), it was observed that the differences between TADF and DADF can be minimal for low loads pulled by large and well-trained oxen. In contrast, time averages can offer bigger and unpredictable values for large jerky loads pulled by small and inexperienced animals (45). This could be because animals slow down when confronted with a large draught force.

Interestingly, donkeys can cover a distance of 20.5 km while working continuously until exhaustion with a speed chosen by themselves and pulling a load equivalent to 21% of their body weight (46). When the draught load increases from 500 to 600 and 700 kg in working donkeys weighing 159 kg, the work speed begins to decline from 0.97 to 0.81 and 0.70 ms^−1^, respectively, suggesting that speed and applied loads are inversely related (47). Speed is an essential parameter for assessing donkeys’ limits of pulling a load, as a voluntary decrease in speed appears to be a reliable predictor of fatigue in donkeys (46). Therefore, donkey owners and working equine welfare advocates can use this indication to determine donkey loading limitations. Though donkeys are generally referred to as “pack animals,” research has shown that they are highly efficient at pulling loads. Donkeys can pull about 2.7 times of their live weight. However, suppose the donkey is subjected to continuous and long working hours (almost 6 h). In that case, it is recommended to keep the load about double of their live weight to safeguard the donkey’s welfare (47).

Donkeys are more efficient in carrying and pulling loads than oxen and buffaloes. The energy costs of pulling loads (5–18 kg) by donkeys are 26.5, 15.3, and 6.2 J m^−1^ kg^−1^ at 0, −10%, and − 15% slope (48). The energy cost is lower at a higher downward slope, as donkeys may be fully utilizing the potential energy of their body weight and the load, probably reducing the energy cost of locomotion. However, as the work rate increases, the efficiency of performing work decreases in donkeys. Their response to exercise is strikingly similar to that of the horse in several parameters, including the extent of its aerobic capacity and locomotor efficiency (49). However, due to the adaptations of the Thoroughbred and Standardbred for high-intensity work, research involving non-racing breeds of horse may be more relevant for studying and predicting donkey performance. For example, it is important to note that there is a difference between walking patterns of donkeys and horses, with some evidence that they walk with a lateralized stride pattern nearing a pace (50) rather than a true four-time walk which is usually observed in horses (51). The donkeys had a shorter stride time (0.87 s), stance time and swing time (forelimbs only) in comparison to previous studies in ponies walking with same speed (1.25 ms^−1^), but have similar swing phases in the hindlimbs (50, 51). This could indicate that the biomechanical consequences of loading investigated in horses cannot be simply translated to donkeys. It is also recognized that donkeys and horses differ physiologically, so results from horse research may not be applicable to donkeys (7, 17, 52).

Standardbred racehorses pulling a small carriage (a “sulky”) suffer different injuries to Thoroughbred racehorses racing with a rider. Musculoskeletal injuries are the leading cause of reduced training days and racehorse wastage (31), and so injuries in Standardbred and Thoroughbred racehorses and the differences between them are well-studied. Many researchers have concentrated on their unique concerns, such as injuries to the middle carpal joint (53) and fractures of proximal sesamoid bone (54). This may be due to uncommon catastrophic accidents during competitions, therefore there are fewer concerns about the safety of races associated with load pulling (31). For example, tibial stress fractures are rare in load pulling racehorses, as are lateral condylar fractures and biaxial proximal sesamoid bone fractures (31). Improved gait mechanics and efficiency can be achieved with age and training in load-pulling racehorses (55). The lack of catastrophic injuries such as suspensory breakdown in load pulling racing may be related to slower speeds and a more caudal position of the center of mass compared to Thoroughbred racehorses (56, 57). Age, gender, driver, racing speed, racing intensity, racing shod, and medical treatment are potential risk factors concerning musculoskeletal injuries (31). However, Injuries in working horses and donkeys pulling loads are less well studied.

In addition to the impact of loading on gait biomechanics, it is important to consider the effects of fatigue. Fatigue increases injury risk (37), and is likely to impact on the welfare of working equids pulling loads for long hours. Generally, increased stride length and stance time are seen in horses due to locomotor fatigue. Johnston et al. (37) tested Standardbred horses, fatigue increases stride length (from 3.74 to 3.87 m) in response to a pulling load of 34 kg, working with a speed of 7 ms^−1^, and stance time reverts back to a non-loaded value (from 0.157 to 0.165 s) (37). Swing time does not change with loading, but does alter with fatigue, increasing from 0.370 to 0.394 s (37). Finally, as a result of increased joint excursion during the stance phase, the forelimb and hindlimb become more flexed due to fatigue (36). Heavier loading may cause a shorter vertical displacement and a stronger forward impulse from hindlimbs to the horse’s body (36, 37).

## 3. Physiological effects of loading

**Figure 5:**
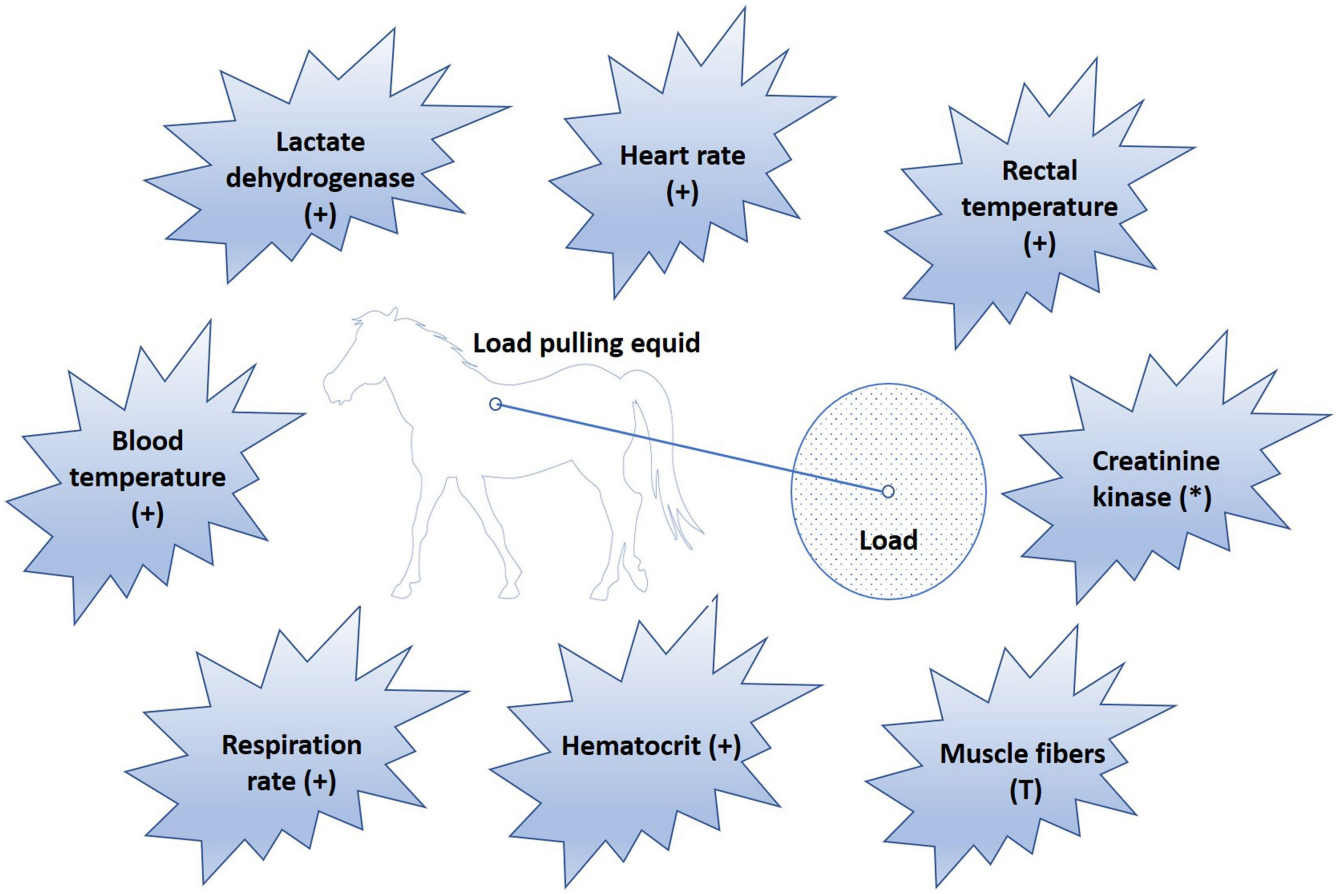
Physiological parameters studied in relation to load pulling in equids. Sign (+) indicates, value of the respective parameter increases in response to work. Symbol (*) indicates, parameter value increases in untrained equids but remains normal in trained working animals. Sign (T) indicates that as work intensity increases, muscle fibers are recruited in the order from type I to IIA, and IIB.

Physiological indicators such as blood temperature (58, 59), rectal temperature (39, 42, 46, 60, 61), heart rate (30, 39, 40, 42, 46, 47, 49, 61, 62), respiration rate (30, 39, 42, 46, 61), hematological profile (30, 40, 49, 61, 62), muscle fiber composition (39, 40, 63), creatinine kinase (62, 64, 65), lactate dehydrogenase (42, 62, 63), alanine aminotransferase, aspartate aminotransferase, alkaline phosphatase, citrate synthase, and 3-hydroxy acyl-CoA dehydrogenase (42, 63, 64), have been investigated in relation to the load pulling capabilities of equids (Figure 5). However, the conditions under which this work has been done have been highly variable, so generalization of the results is difficult. Moreover, there is no research available on working equids in field conditions in LMICs, which often have high temperatures, high humidity, and rough terrain. The physiological impact of load pulling in field conditions would be different from ideal indoor conditions.

Additionally, donkeys are frequently utilized for load pulling, despite the fact that the majority of study on the impact of pulling load has been undertaken on horses. This is significant because donkey physiology frequently differs from horse physiology (7, 52). Compared to horses, donkeys have a lower resting body temperature (36.5–37.7°C), higher resting heart rate (31–53 beats/min.), and higher respiration rate (13–31 breaths per min) ranges (17, 52). Donkeys have fewer erythrocytes (i.e., a lower packed cell volume), but they are larger than those in horses (7). Therefore, there is a need of detailed research on the impact of load pulling on donkey’s physiology.

Compared to many other species, the horse has an obvious disadvantage for heat dissipation as it has a high metabolic capacity, but a small surface area, especially since sweat evaporation is the primary method of heat dissipation (66). During work, temperature increase is more rapid with 1 min of exercise at VO2max as compared to 62% VO2max (being 38.3°C and 37.9°C, respectively). However, blood temperature at fatigue remains the same for both VO2max and 62% VO2max, that is, 41°C (59). This is important because if an animal is fatigued while pulling a load, the work intensity does not matter in relation to metabolic heat production. In trotting horses (9 ms^−1^), rectal temperature increases from 37.9°C to 39.2°C irrespective to level of draught forces (0.1, 0.2, and 0.3 kN) (39). Interestingly, horses working with a speed of 2 ms^−1^, an increase in draught force from 0.33 kN to 0.78 kN do not result in a significant increase in rectal temperature (38.3°C–38.5°C) (60). However, in competition horses, pulling 2.5 times their body weight over a 60-m hard beach sand track for 1.2 min, rectal temperature increases from 37.8°C to 38.4°C (61). In horses (weighing 648 kg) working continuously for a longer period (4 h), rectal temperature increases (from 37.7°C to 38.5°C) with exercise consisting of 0.59 kN draught force over a distance of 26.63 km with a speed of 2.11 ms^−1^. However, this change in rectal temperature recovers within 2 h of rest after the end of exercise (42). In donkeys, pulling load equivalent to 21% of their body weight (235 kg), trotting with a speed of 2.5 ms^−1^ for 30 min, rectal temperature increases from 37.2°C to 39.3°C. However, this change in rectal temperature does not recover even after 1 h of rest after the end of exercise (46). This may be due to the faster metabolic rate in donkeys than horses (64), but this has yet to be investigated in relation to load pulling. These studies indicate that work speed may be more important than draught force in influencing body temperature, but a direct comparison between work speed and draught force in load pulling equids is never made.

Heart rate is a stress quantifying metric used to define stress levels under continuous work. It is known that heart rate is a vital parameter for instant evaluation of health status, training load, and adaptability of equids (7, 31). Workload is reflected in the heart rate reaction to exercise, which increases linearly with the larger the draught load (37, 39, 40, 49). Heart rate increases (from 41 to 76 bpm) with exercise consisting of 0.59 kN draught force over a distance of 26.63 km with a speed of 2.11 ms^−1^ in horses of 648 kg body weight. This change in heart rate does not recover even after 2 h of rest after exercise (42). However, in competition horses, pulling 2.5 times their body weight over a 60-m hard beach sand track for 1.2 min, heart rate increases from 40 to 105 bpm (61). Therefore, the working terrain friction coefficient (FC) is critical for calculating equid load pulling capacity (67). Heart rate is also an important indicator of the speed of work in addition to draught force (40). While explaining the impact of load pulling on working equids, work speed may have greater importance than actual draught force. As the working speed of horses increases from 6 ms^−1^ to 9 ms^−1^ with a constant draught force (0.2 kN), heart rate increases linearly from 167 to 203 bpm (39). Therefore, it is essential to consider both pulled weight and speed of working for accurate quantification of load pulling abilities of equids.

Respiration rate is a function of speed and draught force similar to heart rate, i.e., respiration rate increases with the increase in draught load, but this change in respiration rate is less when compared with the effects of mounted load on respiratory responses (68). Work consisting of 0.59 kN draught force over a distance of 26.63 km at a speed of 2.11 ms-1, respiration rate in horses (weighing 648 kg) increases (from 24 to 52 breath/min). This change in respiration rate does not recover even after 2 h of rest after exercise (42). This could be due to the animal being severely overheated, as respiration should return to normal if the horse is used to working. However, in competition horses, pulling 2.5 times their body weight over a 60-m hard beach sand track for 1.2 min, respiration rate increases from 32 to 56 breaths/min (61). Hence, the working terrain (FC) is important for calculating equids’ load-hauling capabilities (67). In trotting horses (9 ms^−1^), a greater increase in respiration rate, from normal to 110, 119, and 104 breath/min, has been seen with a less draught force of 0.1, 0.2, 0.3 kN (39). Even though the draught force is low, this increase in respiration rate is caused by work speed. As the working speed of horses increases from 6 ms^−1^ to 9 ms^−1^ with a constant draught force (0.2 kN), the respiration rate increases from 94 to 119 breath/min (39). Therefore, for accurate estimation of load pulling capacities of equids, it is necessary to account for both hauled weight and working speed.

The hematological profile is essential in determining physiological changes occurring in equids (65). Hematological parameters change due to exercise in both horses and donkeys (17). Both draught weight and work speed are proportional to changes in red cell volume. The red cell volume is thought to measure the horse’s oxygen transport capacity (40, 69). Furthermore, the horses hauling the heaviest weights and trotting at the maximum trotting speeds have the highest red cell volume (40). In a team of two horses (one colt and one stallion), pulling draught load of 0.93 kN draught force while working together for 150 min, hematocrit (Hct%) increases from 34.8 to 42.6% and from 37.8 to 45.5% in colt and stallion, respectively (62). In donkeys working with a speed of 1.8 ms^−1^ and a draught force of 0.4 kN for 25 min, Hct% increases from 39 to 48.6% (49). This implies that aerobic capacity is necessary for both draught-loaded exercise and work speed, both of which are crucial factors for quantifying the impact of load hauling on equids.

Horses are believed to get stronger, increase muscle volume, and have enhanced endurance due to load-related workouts (39). The need for force grows as the draught load increases, and the rate of energy expenditure in the muscles may surpass the horse’s maximum rate of oxygen supply, so the oxidative capacity of the muscles is important (40). In muscles, type I fibers have a low ATPase activity, and a high oxidative capacity, and a low glycolytic capacity. Type IIA fibers have a high myosin ATPase activity, and a high oxidative and glycolytic capacity. Type IIB fibers have a high myosin ATPase activity, and a low oxidative capacity, and a high glycolytic capacity (70). As work intensity increases, fibers are recruited in order, from type I to IIA to IIB. Type I and a significant proportion of type II fibers are recruited at rapid trotting speeds (40). Type I and IIA muscle fibers (in the gluteus medius, longissimus, and brachiocephalicus muscles) increase, while type IIB muscle fibers decrease in response to a 12-week draught loaded exercise test (0.33 kN draught force, with speed ranging from 5.5 to 8 ms^−1^ for 12 min) (63). Compared to draught horses, Thoroughbreds can exert the same draught forces and reach double the speed, external power, and oxygen consumption. Thoroughbred horses’ maximum oxygen consumption is reported to be roughly twice that of draught horses, showing adaptations to high-intensity activity (38). Compared to Thoroughbred horses, draught horses’ peak efficiency occurs at lower speeds, demonstrating adaptations to high-force and low-speed activities. The disparities in force, oxygen consumption, and peak efficiency speed between draught horses and Thoroughbreds are most likely due to distinct locomotor muscle contraction velocities (38), and maybe due different muscle fiber types (type I, type IIA, and IIB). These disparities in locomotor muscle contraction velocities, and the order in which muscle fiber types are recruited, have yet to be explored in donkeys and mules.

Creatinine kinase is a muscle-specific enzyme with a half-life of 2 h in the blood (42). In horses, a spike in serum creatine kinase enzyme activity is a helpful diagnostic of post-exercise muscle soreness and muscle injury (17). Since its rise in plasma activity is greater in untrained horses than in trained horses, measuring creatinine kinase concentrations could be a helpful fitness indicator (42). At a maximum load that a horse can pull over a distance of 14 feet, during heavy horse pull competition at Calgary Stampede (71), creatine kinase enzyme activity increases from 174 to 225.5 IU/L (64). However, in a team of two horses (one colt and one stallion), pulling draught load of 0.93 kN draught force while working together for 150 min, creatine kinase enzyme activity increases from 62.1 to 101 U/L and from 127 to 167 U/L in colt and stallion, respectively (62). On the second day of work, it may recover to its baseline levels (42), after which the values can remain within the normal range (for colts, 62 ± 52 U/L; for stallions, 127 ± 67 U/L) (62). This would show that the equids have adapted to load-pulling work. Hence, changes in creatinine kinase activity in the blood may be a reliable indication of an equids’ aptitude for load-pulling work.

The lactate dehydrogenase enzyme is commonly found in muscles (63). Although it is usually believed that an increase in the concentration of muscle enzymes in plasma indicates muscle damage, given the slight variations in these enzymes’ values within normal ranges described for horses, it is possible that the changes in these enzymes’ values are due to changes in the permeability of the muscular cell membrane (42). In a team of two horses (one colt and one stallion), pulling draught load of 0.93 kN draught force while working together for 150 min, lactate dehydrogenase activity in blood increases from 634 to 785 U/L and from 604 to 646 U/L in colt and stallion, respectively (62). No change in lactate dehydrogenase activity occurs (in the gluteus medius, longissimus, and brachiocephalicus muscles) in response to a 12-week draught loaded exercise test (0.33 kN draught force, with speed ranging from 5.5 to 8 ms^−1^ for 12 min) (63). However, the length and intensity of exercise positively correlate with the rise in plasma enzyme activity after exercise. This rise can be mitigated with proper training (62).

## 4. Changes in biochemical indicators

Biochemical indicators such as blood lactate (38, 39, 49, 62, 72, 73), blood oxygen, blood carbon dioxide level (42, 59), blood glucose (42, 60, 62, 64, 73), and adenosine tri phosphate has been investigated in relation to load pulling (59, 60). Moreover, sodium, chloride, potassium (61, 64), plasma protein (61, 64, 74), uric acid, urea (61, 62, 64, 73), plasma triacylglycerols, free fatty acids, and cholesterol (60, 62, 73) have also been investigated in relation to load pulling capabilities of equids. However, these studies have used different parameters in different conditions and the number of studies is insufficient for each parameter to provide a comprehensive understanding of the effect of draught load on the biochemical parameters and quantification of draught load pulling abilities of equids. Therefore, it may be important to quantify their load pulling ability in standardized working conditions.

The lactate concentration in the blood is a reliable indicator of the load effect (17) because the commencement of anaerobic metabolism is signaled by increased blood lactate levels, which is related to a reduced ability to maintain a given exercise level in equids (49). In response to load pulling, blood lactate levels rise sharply (40), and it increases exponentially with an increase in draught force and velocity (40, 68). In working horses (9 ms^−1^), as the draught force increases from 0.1kN to 0.3kN, plasma lactate rises from 3.8 to 10.8 mmoL/L. Similarly, with the increase in work speed from 6 ms^−1^ to 9 ms^−1^, with a constant draught force (0.3 kN), plasma lactate increases from 4.5 to 10.8 mmoL/L (39). If we compare Thoroughbred and draught horses, plasma lactate increases from resting level (0.8 mmoL/L) to 7.3, 12.4, 11.4, 10.5, 6.7 mmoL/L and from resting level (0.8 mmoL/L) to 4.4, 12, 12.6, 7.3, 12.7 mmoL/L in Thoroughbred and draught horses, with draught force equals to 0, 5, 10, 15, and 20% of their body weight, respectively (38). This demonstrates the metabolic difference between Thoroughbred and draught horses at lower and higher levels of load pulling, although there is no doubt that both breeds use anaerobic metabolism at various levels. Furthermore, when comparing a young and experienced horse, the older horse has a lesser increase in blood lactate as the adaptation to pulling load occupation develops with the passage of time (62).

In horses, skeletal and cardiac muscle oxygen requirements rise in proportion to their metabolic needs. The main limiting elements in intensive muscular exertion are oxygen-carrying functions of the circulatory system and oxygen use in muscles (30). In horses (weighing 648 kg) working continuously for a longer period (4 h), arterial oxygen level (*p*O_2_) decreases from 103 mmHg to 93.8 mmHg, and venous *p*O_2_ increases from 46.8 mmHg to 51 mmHg. Whereas, arterial carbon dioxide level (*p*CO_2_) increases from 32.9 mmHg to 35.4 mmHg, and venous *p*CO_2_ decreases from 36.5 mmHg to 35.3 mmHg with exercise comprising of 0.59 kN draught force over a distance of 26.63 km with a speed of 2.11 ms^−1^ (42). The oxygenation of arterial blood during exercise decreased, limiting oxidative metabolism (59). Although an increase in venous *p*O_2_ appears to reflect a decrease in tissue oxygen consumption, it could just be a redirection of blood flow to places like the skin to aid heat dissipation. As they took jugular blood samples for venous *p*O_2_ (30), which is venous drainage from the head and neck areas where oxygen use may be reduced during exercise, causing an increase in venous *p*O_2_ during work.

The use of glucose in the muscle during load-pulling exercises is determined by the weight of draught load and the duration and speed of work (42). The most common reaction of horses to pulling load at low speeds for long periods is either no change or reduced blood glucose concentrations (42). In horses working with a speed of 2 ms^−1^ and a draught force of 0.33 kN, blood glucose level decreases from 5.6 to 4.4 mmoL/L (60). Interestingly, at a maximum load that a horse can pull over a distance of 14 feet, blood glucose level remains unchanged during heavy horse pull competition at Calgary Stampede (64, 71). However, in mules (320–380 kg bodyweight), working under a draught load equals 10% of their body weight for 2 h, blood glucose level decreases from 5.417 to 4.917 mmoL/L (73).

Adenosine triphosphate (ATP) is also affected by load-pulling inside horses (59). Interestingly, no marked changes occurred in the levels of muscle ATP in horses working with a speed of 2 ms^−1^ with either a draught force of 0.33 kN or 0.78 kN (60). In an identical fashion, no marked changes occur in the level of muscle ATP in response to exercise at 62% of VO2max. However, ATP contents decrease significantly in response to exercise at VO2max (59). Intense exercise, demanding more oxygen and energy, can reduce ATP level, and it is not affected by less energy-demanding work.

Fluid and electrolyte losses can compromise optimum exercise performance (75, 76). At a maximum load that a horse can pull over a distance of 14 feet, during heavy horse pull competition at Calgary Stampede (71), plasma sodium, chloride, and potassium decreases from 129.5 to 125.5, 95 to 92, and 3.3 to 2.9 mmoL/L, respectively (64). In contrast, in another study, pulling exercise caused a short-term elevation in sodium and chloride, which rapidly returned to resting values within 15 min in horses (61). During exercise and recovery, the renin-angiotensin-aldosterone axis (RAA) is linked to the acute and chronic defense of blood pressure, plasma volume, along with fluid and electrolyte balance (74–76). Furthermore, acute hypovolemic stress activates the RAA axis (74), and high aldosterone and arginine vasopressin concentrations are associated with exercise in horses (75, 76). Exercise has little effect on renin levels, although it does increase aldosterone and arginine vasopressin levels (74).

In horses, plasma protein contents are affected by load pulling work (61), but it is likely to be due to dehydration level, not due to duration or intensity of work. In horses, pulling 2.5 times their body weight over a 60-meter hard beach sand track for 1.2 min, total plasma protein increases from 7.8 g/dL to 8.5 g/dL, and plasma albumin increases from 3.5 g/dL to 4 g/dL (61). Interestingly, at a maximum load that a horse can pull over a distance of 14 feet, during heavy horse pull competition at Calgary Stampede (71), total plasma protein, albumin, and globulin remained the same before and after the competition (64). However, the level of total plasma protein and albumin critically depends on the hydration status of horses (74, 77). If the horse is dehydrated, he will have a higher level of total plasma protein contents per unit volume of plasma.

Generally, blood nitrogen contents (uric acid and urea) increase after load-associated work in equids (17, 61, 62, 73). In a team of two horses (one colt and one stallion), pulling draught load of 0.93 kN draught force while working together for 150 min, plasma uric acid increases from 0.014 to 0.041 and from 0.017 to 0.026 mmoL/L in colt and stallion, respectively (62). As far as plasma urea level is concerned, in horses, pulling 2.5 times their body weight over a 60-meter hard beach sand track for 1.2 min, total plasma urea contents increase from 7.2 mmoL/L to 9.5 mmoL/L (61). However, at a maximum load that a horse can pull over a distance of 14 feet, during heavy horse pull competition at Calgary Stampede (71), plasma urea contents remained the same before and after the competition (64). Interestingly, in mules (320-380 kg bodyweight), working under a draught load equals 10% of their body weight for 2 h, serum urea increases from 8.7 to 12.8 mmoL/L (73). Plasma nitrogen concentration is considered a parameter of overtraining in humans (78). Therefore, a rise in plasma nitrogen contents could be a concern and an important indicator of load pulling limits in equids.

Plasma triacylglycerols and free fatty acids (FFA) are crucial biochemical measures to understand the impact of pulling a load in equids because the changes in plasma triacylglycerol levels reflect the intensity of exercise (62, 79), and plasma FFA represents important oxidative metabolic substrates, especially when pulling load for long periods. In a team of two horses (one colt and one stallion), pulling draught load of 0.93 kN draught force while working together for 150 min, plasma triacylglycerol increases from 0.28 to 0.66 mmoL/L and from 0.31 to 0.53 mmoL/L in colt and stallion, respectively (62). This increase of triacylglycerol is specific for exercising horses; it was not observed in rodents or human beings. In horses working with a speed of 2 ms^−1^ and a draught force of 0.33 kN, plasma free fatty acids (FFA) increase from 300 to 790 μmoL/L. During post-exercise resting intervals, FFA levels increased more than during walking intervals (60). It is important to remember that, during load pulling work, horse FFA usage varies depending on draught resistance, velocity, and duration of activity (60). Furthermore, In mules (320-380 kg bodyweight), working under a draught load equals 10% of their body weight for 2 h, blood cholesterol level decreases from 2.570 to 2.239 mmoL/L (73), which may be due to their utilization during load pulling work. However, these studies were performed under different conditions; a standardized approach may be used to compare these parameters better and understand the impact of pulling load on equids.

## 5. Behavioral measures and indicators of stress

**Figure 6:**
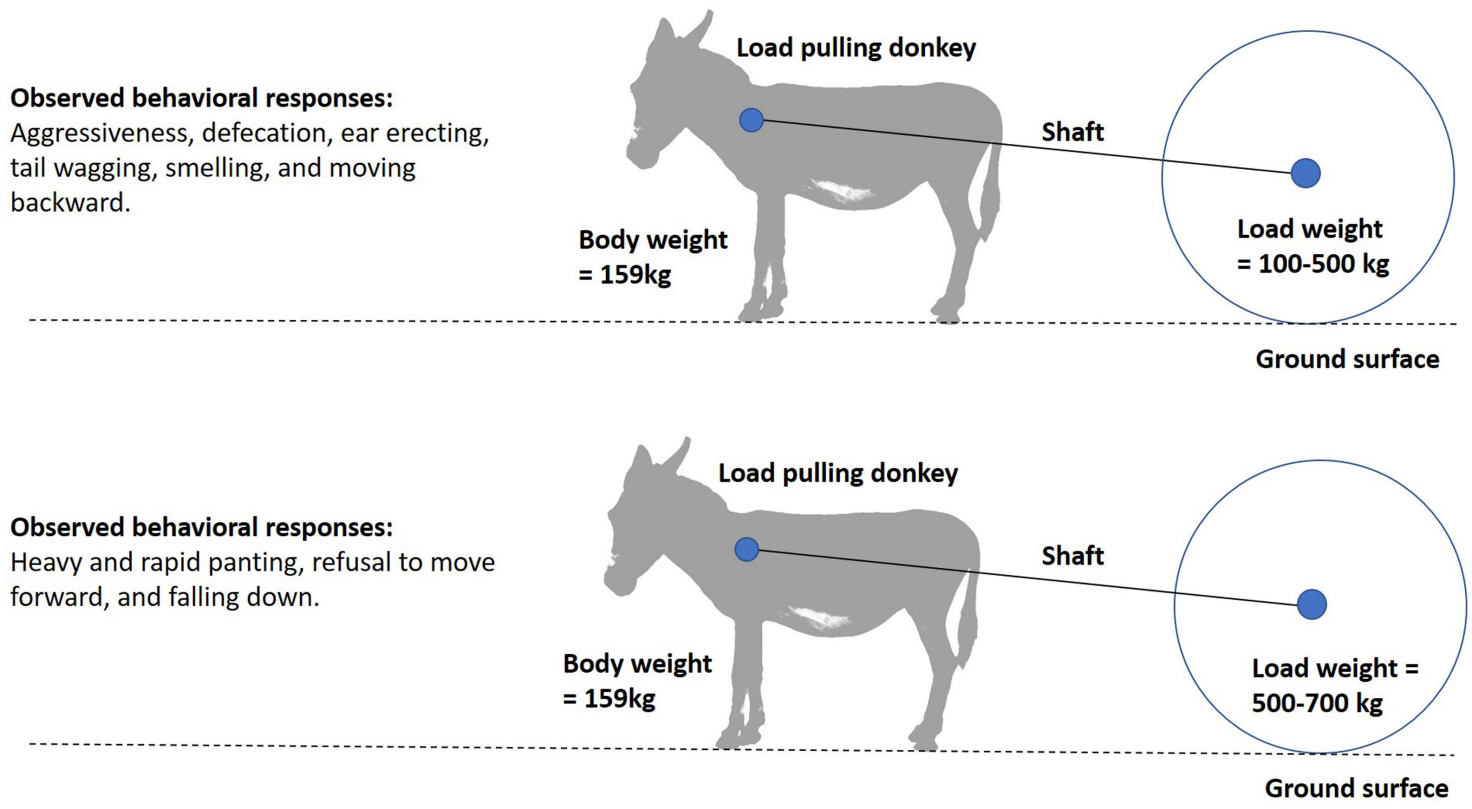
Behavioral responses of load pulling donkeys in response to different load weights (47).

The use of behavioral cues to evaluate the impact of draught load in equids is still in its early stages. Assessment of donkeys’ stress responses are always conducted based on irregular behavioral phenomena that may be difficult to interpret (80, 81). Behavioral responses are the first line of defense to environmental challenges and stress. In donkeys, signs of fatigue include unwillingness to continue, uncoordinated legs and excitement after work (47). Behavioral problems like hyperesthesia, depression, non-responsiveness, avoidance, aggressive response, and avoiding chin contact have been observed in donkeys pulling heavy brick kiln load (23, 82). An improved general attitude and reaction to observers are associated with an improved body condition. As a consequence, it is important for the owners of working donkeys to pay attention to changes in their body condition in order to avoid compromising their welfare (12). Draught load associated changes in the donkey’s behavior are shown in Figure 6. While ridden changes in behavior due to loading have been investigated in horses (83), draught load associated changes in horse and mule behavior have yet to be investigated.

The speed of draught load pulling donkeys is also an important behavioral measure, as it has an inverse relation with the weight of load (47). Therefore, speed is an important parameter for assessing donkeys’ limits of pulling a load, as a voluntary decrease in speed appears to be a reliable predictor of fatigue in donkeys (46). Working donkey owners should pay attention to the speed of walking donkeys and take necessary measures to avoid compromising donkey welfare and performance during their routine work.

Equine stress must be measured to assess an equid’s emotional state and welfare. An ethogram has been used to assess musculoskeletal pain-associated behaviors in horses (84), which may only be helpful when used by trained assessors (85). More recently, a grimace-scale for assessing pain has been developed for use in donkeys (86), although this has not yet been used in the field with working donkeys. While no ethogram has been designed for load-pulling horses, mules, and donkeys, other measures that are easy to assess in the field are becoming available. Recently it has been reported that eye blink rate is a cheap, simple, and immediate indicator of acute equine stress (87–89). As it has been seen that in the presence of a stressor (presentation of the clipper), blink rate first decreases (7 blinks/min) and then go higher (13 blinks/min) than the resting blink rate (10 blinks per min) in stable horses (87). Therefore, it may aid in pulling load equine welfare assessment (87–89). Traditional stress measurement techniques, such as heart rate, heart rate variability (HRV), cortisol level, and more recently, changes in eye temperature (17, 90, 91), need special equipment which are not readily available in the equine’s working environment. However, the use of spontaneous blink rate for stress assessment needs to be investigated in working equids.

In animal science, heart rate variability is a regularly used statistic to quantify a physiological response to stresses. HRV analysis relies on accurate detection of the heart’s electrical activity (90). Heart rate variability is the variation in the time interval between heartbeats. It decreases with heavy riders (20% body weight ratio) as compared to lighter riders (10% body weight ratio) (92). There is no study available assessing HRV association with pulling load for working horses, mules, and donkeys.

Cortisol is not a good measure of work and load-related stress because it may also be significantly affected by diet, genetic factors, environment, and characteristics associated with individuals (93, 94). Generally, changes in plasma cortisol levels reflect the intensity of exercise (62), stress level, including exercise-induced stress in equids (49). In a team of two horses (one colt and one stallion), pulling draught load of 0.93 kN draught force while working together for 150 min, plasma cortisol increases from 382.5 to 785 and from 234.7 to 482.5 nmoL/L in colt and stallion, respectively (62). Moreover, in donkeys (weighing 183 kg), plasma cortisol increases from 76 to 399 nmoL/L with a draught force of 0.4 kN for 25 min with a speed of 1.8 ms^−1^ (49). However, salivary cortisol measurement is far superior to plasma cortisol measurement for assessing stress and hypothalamus-pituitary–adrenal activity because it avoids the need to account for between-subject differences in cortisol binding globulin or within-subject alterations (17). Here, the difference in cortisol levels between horses and donkeys could be due to the difference in duration and intensity of exercise. Moreover, it is known that donkeys’ response is similar to horses as far as plasma cortisol level is concerned (49).

## 6. Conclusion

One of the many issues that may jeopardize working equine welfare is pulling overly heavy loads. Much research has been done over the last four decades to understand the effect of load pulling on horse performance, but the effect on donkeys and mules has received less attention. As a consequence, we have no idea how much weight a working equid can pull. Load pulling affects a wide range of biomechanical, physiological, biochemical, and behavioral characteristics in equines, and more research is needed to advance our understanding of these factors, particularly in donkeys and mules. Quantified load pulling limits could then be used by non-governmental organizations (NGOs), policymakers, and other stakeholders working with vulnerable communities and working equids to limit excessive load pulling and improve animal welfare.

## Author contributions

SB and RP were involved in the preparation of the manuscript, gave final approval of this manuscript, read, and agreed to the published version of the manuscript.

## Funding

This project was funded by City University of Hong Kong (Grant Number 9610463).

## Conflict of interest

The authors declare that the research was conducted in the absence of any commercial or financial relationships that could be construed as a potential conflict of interest.

## Publisher’s note

All claims expressed in this article are solely those of the authors and do not necessarily represent those of their affiliated organizations, or those of the publisher, the editors and the reviewers. Any product that may be evaluated in this article, or claim that may be made by its manufacturer, is not guaranteed or endorsed by the publisher.
